# A combined carbohydrate antigen 125 and lymphocyte-to-monocyte ratio score stratifies time-window-specific recurrence after pancreatic cancer surgery

**DOI:** 10.3389/fmed.2026.1971334

**Published:** 2026-10-08

**Authors:** Bin Yi, Xiance Tang, Xuejiao Chen, Qingfeng Tian

**Affiliations:** 1 School of Public Health, Zhengzhou University, Zhengzhou, China; 2 Follow-up Office, Henan Cancer Hospital, Zhengzhou, China; 3 Scientific Research and Foreign Affairs Office, Henan Provincial People’s Hospital, Zhengzhou, China

**Keywords:** biomarkers, CA125, disease-free survival, early recurrence, lymphocyte-to-monocyte ratio, pancreatic cancer, risk stratification

## Abstract

**Background:**

Early recurrence after pancreatic resection is a major cause of treatment failure in pancreatic cancer. We evaluated whether a score combining carbohydrate antigen 125 (CA125) with the lymphocyte-to-monocyte ratio (LMR) could stratify recurrence risk across clinically relevant time windows.

**Methods:**

This retrospective cohort included 1,618 patients who underwent pancreatic cancer surgery. Recurrence within 6, 12, and 24 months was defined as ER6, ER12, and ER24, respectively, with ER12 prespecified as the primary endpoint. ER12-derived cutoffs were 44.760 U/mL for CA125 and 5.764 for LMR; one point was assigned for elevated CA125 and one for low LMR, yielding a 0–2 score. Discrimination was assessed using receiver operating characteristic curves and pairwise DeLong tests, and disease-free survival was evaluated using Kaplan–Meier analysis.

**Results:**

ER12 occurred in 779 patients (48.1%). For ER12, the CA125-LMR score yielded an area under the curve (AUC) of 0.742 [95% confidence interval (CI), 0.720–0.764], with 91.4% sensitivity and 44.3% specificity. Its AUC exceeded those of CA125 alone (0.698; *p* < 0.001) and the CA19-9-LMR score (0.692; *p* < 0.001), but did not differ significantly from that of LMR alone (0.735; *p* = 0.463). The score showed AUCs of 0.694 for ER6 and 0.809 for ER24; at 24 months, LMR alone performed better (AUC, 0.835; *p* = 0.003). Disease-free survival decreased stepwise across scores 0, 1, and 2 (log-rank *p* < 0.001).

**Conclusion:**

The CA125-LMR score is a simple, interpretable tool for recurrence-risk stratification after pancreatic cancer surgery, with its clearest utility in early and intermediate time windows. External validation is required before clinical implementation.

## Introduction

Pancreatic cancer remains one of the most lethal malignancies worldwide, and its incidence and mortality are projected to increase despite advances in systemic therapy and perioperative care (1, 2). Surgical resection offers the principal opportunity for long-term survival in patients with localized disease, yet postoperative recurrence remains common and limits the benefit of surgery (3, 4).

Early recurrence (ER) is therefore a clinically relevant outcome after pancreatic cancer resection. Definitions vary across studies, with thresholds ranging from 6 to 12 months or longer; large recurrence-pattern studies support a 12-month threshold as a pragmatic primary endpoint, and recent studies have linked very early relapse to aggressive disease and limited surgical benefit (4–7). Evaluating multiple time windows may nevertheless reveal whether biomarker performance changes as the outcome shifts from very early relapse to later recurrence.

Preoperative risk-stratification approaches include clinical nomograms, physiobiological scores, imaging and radiomics models, Bayesian frameworks, serum biomarker combinations, and routinely collected metabolic or clinicopathological factors (8–22). Many models are difficult to implement routinely, and their performance may depend on the selected recurrence horizon. Simple biomarker scores based on routinely available blood tests remain attractive when their intended use and limitations are clearly defined.

Serum carbohydrate antigen 19-9 (CA19-9) is the most widely used biomarker in pancreatic cancer. Yugawa et al. combined CA19-9 with LMR to predict 12-month recurrence after resection (23). However, CA19-9 is affected by biliary obstruction, inflammation, and Lewis antigen status, and it may be uninformative in non-producers (24). These limitations support evaluation of complementary tumor markers.

CA125, the circulating epitope of MUC16, has received increasing attention in pancreatic cancer. Clinical studies have linked elevated CA125 with metastatic burden, poor prognosis, and adverse outcomes after pancreatic cancer surgery, while mechanistic work suggests that MUC16 expression may contribute to invasive biological behavior (21, 25–29). In parallel, systemic inflammatory and immune-nutritional indicators, including LMR, have been repeatedly associated with outcomes in pancreatic cancer (30–38). These findings provide a rationale for combining CA125 with LMR as a two-domain biomarker score reflecting both tumor burden and host response.

We developed an ER12-derived CA125-LMR score and evaluated its discrimination for recurrence within 6, 12, and 24 months. We also compared the score with CA19-9, CA125, LMR, and a CA19-9-LMR score; examined disease-free and overall survival; and assessed the sensitivity of results to time-window-specific cutoffs. We hypothesized that the relative performance of tumor and inflammatory biomarkers would vary by recurrence horizon.

## Materials and methods

### Study design and patients

This retrospective observational cohort study is reported in accordance with the STROBE statement (39). We collected data from hospitalized patients with pathologically confirmed pancreatic ductal adenocarcinoma (PDAC) who underwent pancreatic resection at Henan Cancer Hospital between January 2015 and December 2023. Patients were eligible if they (1) had pathologically confirmed pancreatic ductal adenocarcinoma (PDAC) and received inpatient care; (2) underwent pancreatic resection; (3) had no serious immunologic, infectious, or hematologic conditions likely to affect systemic inflammatory markers, including human immunodeficiency virus infection, syphilis, or leukemia; (4) had no other malignant tumors; and (5) had complete clinical and follow-up data. Patients were excluded if they did not undergo the relevant surgical treatment or declined study follow-up. The study protocol was reviewed and approved by the Medical Ethics Committee of Henan Cancer Hospital (approval no. 2026-266-001). The requirement for written informed consent was waived owing to the retrospective design of the study. Among the 1,618 patients, 126 (7.8%) had AJCC stage IV disease, in whom pancreatic resection was combined with synchronous metastasectomy and/or metastasis-directed ablation or other local therapy for limited metastatic disease within an individualised multidisciplinary treatment programme; no patient underwent palliative bypass surgery alone.

### Definitions of recurrence and survival endpoints

The prespecified primary binary endpoint was recurrence within 12 months after surgery (ER12), and the primary time-to-event endpoint was disease-free survival (DFS). ER6 and ER24 were generated analogously and were coded as recurrence documented within 6 and 24 months, respectively. Follow-up information, including recurrence or metastasis and vital status, was obtained by hospital staff through regular telephone follow-up and review of institutional medical records. After surgery, patients were generally assessed at approximately 3-month intervals during the first two years and every 6 months thereafter. Telephone follow-up served to identify potential events and to remind patients of the surveillance schedule; recurrence was defined according to the Diagnosis and Treatment Guidelines for Pancreatic Carcinoma issued by the National Health Commission of the People's Republic of China (40): postoperative local recurrence, peritoneal implantation metastasis, and distant metastasis were all defined as recurrence after pancreatic cancer surgery. Local recurrence referred to regrowth of the tumour in the original surgical field, including the pancreatic bed, the regions around the superior mesenteric artery and vein and the coeliac trunk, the portal–splenic vein confluence, and the anastomotic regions. Peritoneal implantation metastasis referred to implantation of tumour cells on intra-abdominal serosal surfaces, including the greater omentum, the mesentery, the parietal and visceral peritoneum, and the surfaces of the liver, spleen, and diaphragm. Distant metastasis referred to haematogenous or lymphatic spread of the tumour to organs or tissues outside the abdominal cavity, most commonly the liver parenchyma, the lungs, bone, and brain. Recurrence was determined primarily by imaging evidence, such as contrast-enhanced CT or MRI, combined with serial increases in serum CA19-9; a proportion of cases were confirmed by cytology or pathology. For patients with recurrence, recurrence time was calculated in months from surgery to the first documented recurrence or metastasis; for patients without recurrence, it was calculated from surgery to the last follow-up. Patients classified as non-recurrent at a given landmark had documented follow-up through that time point. DFS was defined as the interval from surgery to the first documented recurrence or metastasis or death from any cause, whichever occurred first; patients who were alive and disease-free were censored at the last follow-up. Overall survival (OS) was calculated from surgery to death from any cause, with survivors censored at the last follow-up. Follow-up completeness, event status, and all derived time-to-event variables were verified before analysis. In each recurrence window, patients who died without documented recurrence were distinguished from censored patients; in this cohort, all 371 patients who died within 12 months of surgery had documented recurrence within 12 months (competing events, *n* = 0).

The three recurrence windows addressed distinct clinical horizons. ER6 represented very early relapse, ER12 was prespecified as the primary endpoint because it is commonly used after pancreatic cancer resection (5, 23), and ER24 was used to determine whether the ER12-derived score retained discrimination for later recurrence. These analyses were prognostic and were not intended to establish causal mechanisms.

### Biomarkers and score construction

The laboratory value closest to surgery was used for each preoperative test. CA125 and CA19-9 were measured in U/mL, and absolute lymphocyte and monocyte counts were measured in ×10^9^/L. LMR was calculated as the absolute lymphocyte count divided by the absolute monocyte count. For patients who underwent biliary drainage, post-drainage results were selected. Measurements obtained during acute infection or clinically apparent inflammation were excluded. These selection rules were applied using the laboratory and clinical records available at the time of data collection Other evaluated markers included carcinoembryonic antigen (CEA), prognostic nutritional index (PNI), neutrophil-to-lymphocyte ratio (NLR), platelet-to-lymphocyte ratio (PLR), and C-reactive protein-related indices.

The CA125-LMR score used optimal ER12 cutoffs selected by the Youden index. One point was assigned for CA125 > 44.760 U/mL and one point for LMR < 5.764, producing scores of 0, 1, or 2. A CA19-9-LMR comparator score was constructed using the same rule. Cutoffs were derived in the study cohort; consequently, performance estimates should be interpreted as internally derived and require external validation.

The source dataset included demographic characteristics (age at first admission and sex); treatment variables (chemotherapy and targeted therapy); laboratory variables (C-reactive protein, CA125, CA19-9, CEA, albumin, PLR, LMR, and PNI); and clinicopathological variables (TNM stage, tumor location, tumor size, differentiation, resection margin, perineural invasion, and vascular invasion). Outcome variables comprised recurrence, death, recurrence time, and survival time. The present analyses focused on the variables reported in Table 1 and biomarker comparisons.

**Table 1:** Baseline characteristics according to 12-month recurrence.

Variable	N	Overall	Non-ER12	ER12	*P* value
TNM stage	1,618				<0.001
I		282 (17.4%)	176 (21.0%)	106 (13.6%)	
II		536 (33.1%)	314 (37.4%)	222 (28.5%)	
III		674 (41.7%)	303 (36.1%)	371 (47.6%)	
IV		126 (7.8%)	46 (5.5%)	80 (10.3%)	
C-reactive protein	1,618	7.060 (1.920, 35.340)	4.740 (1.350, 24.700)	10.670 (2.950, 42.910)	<0.001
CA125, U/mL	1,618	28.920 (14.790, 92.770)	20.880 (11.460, 41.320)	50.680 (21.270, 178.200)	<0.001
CA19-9, U/mL	1,618	82.660 (16.350, 295.400)	47.880 (11.920, 228.300)	130.200 (22.630, 366.100)	<0.001
CEA	1,618	3.895 (2.090, 9.460)	2.950 (1.750, 5.430)	5.680 (2.760, 18.370)	<0.001
Albumin	1,618	40.300 (36.600, 43.300)	40.700 (37.500, 43.800)	39.600 (35.300, 42.700)	<0.001
PNI	1,618	47.800 (43.150, 52.000)	48.600 (44.650, 53.100)	46.600 (41.650, 50.950)	<0.001
PLR	1,618	138.755 (102.985, 194.017)	132.530 (100.971, 184.397)	145.631 (104.706, 207.018)	<0.001
LMR	1,618	4.301 (2.918, 7.032)	5.948 (3.674, 9.755)	3.452 (2.530, 4.775)	<0.001
Age, years	1,618	69.450 (47.992, 80.733)	62.530 (39.750, 77.459)	76.153 (57.345, 82.000)	<0.001
Sex	1,618				0.922
Female		729 (45%)	379 (45%)	350 (45%)	
Male		889 (55%)	460 (55%)	429 (55%)	
Differentiation	1,618				<0.001
Undifferentiated/poor		916 (57%)	415 (49%)	501 (64%)	
Moderate/well		702 (43%)	424 (51%)	278 (36%)	
Tumor location	1,618				0.051
Head/uncinate		1,136 (70%)	607 (72%)	529 (68%)	
Body/tail		482 (30%)	232 (28%)	250 (32%)	
Tumor size	1,618				<0.001
≤2 cm		221 (14%)	142 (17%)	79 (10%)	
>2–4 cm		511 (32%)	305 (36%)	206 (26%)	
>4 cm		886 (55%)	392 (47%)	494 (63%)	
Resection margin status	1,618				<0.001
0		1,029 (64%)	588 (70%)	441 (57%)	
1		589 (36%)	251 (30%)	338 (43%)	
Perineural invasion	1,618				0.019
0		1,248 (77%)	667 (79%)	581 (75%)	
1		370 (23%)	172 (21%)	198 (25%)	
Vascular invasion	1,618				0.170
0		1,290 (80%)	680 (81%)	610 (78%)	
1		328 (20%)	159 (19%)	169 (22%)	
Chemotherapy	1,618				0.008
No		202 (12%)	87 (10%)	115 (15%)	
Yes		1,416 (88%)	752 (90%)	664 (85%)	
Targeted therapy	1,618				<0.001
No		1,101 (68%)	523 (62%)	578 (74%)	
Yes		517(32%)	316(38%)	201(26%)	

Data are median (interquartile range) or *n* (%). ER12, recurrence within 12 months; CA125, carbohydrate antigen 125; CA19-9, carbohydrate antigen 19-9; CEA, carcinoembryonic antigen; LMR, lymphocyte-to-monocyte ratio; PNI, prognostic nutritional index; PLR, platelet-to-lymphocyte ratio. TNM stage: I, II, III, or IV; sex: female vs. male; differentiation: undifferentiated/poor vs. moderate/well; tumor location: head/uncinate vs. body/tail; tumor size: ≤2 cm, >2–4 cm, or >4 cm. resection margin status: 0 (negative) vs. 1 (positive); perineural invasion: 0 (negative) vs. 1 (positive); vascular invasion:0 (negative) vs. 1 (positive); chemotherapy: no vs. yes; targeted therapy: no vs. yes.

Categorical variables used prespecified definitions: female vs. male; undifferentiated/poorly differentiated vs. moderately/well differentiated; tumor location in the head/uncinate vs. body/tail; resection margin status 0 (negative) vs. 1 (positive); negative vs. positive perineural and vascular invasion; and no vs. yes for chemotherapy and targeted therapy. Tumor size was categorized as ≤2 cm, >2–4 cm, or >4 cm. Pathological TNM stage was assigned according to the Chinese guidelines for diagnosis and treatment of pancreatic cancer 2018 issued by the National Health Commission of the People's Republic of China, which adopts the eighth edition of the American Joint Committee on Cancer TNM staging system (40). Stage was recorded as I, II, III, or IV. Reference categories were female, and no chemotherapy or targeted therapy, as applicable.

### Statistical analysis

Continuous variables are summarized as medians with interquartile ranges, and categorical variables as counts and percentages. Between-group comparisons used Wilcoxon rank-sum tests for continuous variables and Pearson chi-square or Fisher exact tests for categorical variables, as appropriate. Receiver operating characteristic curves were used to assess discrimination for ER6, ER12, and ER24. Areas under the curve (AUCs) are reported with 95% confidence intervals (CIs); sensitivity and specificity were calculated at the selected cutoff. Correlated AUCs were compared using DeLong tests. DFS and OS were estimated using Kaplan–Meier methods and compared using log-rank tests. The study size was determined by all eligible patients available during the study period; no *a priori* sample-size calculation was performed. The variables used in the reported primary analyses were complete for all 1,618 patients, and no imputation was performed. Tests were two-sided, and *p* < 0.05 was considered statistically significant. Analyses were performed using R version 4.4.2.

The primary analyses applied the fixed ER12-derived CA125 and LMR cutoffs to all three recurrence windows. A sensitivity analysis recalculated the cutoffs separately for ER6, ER12, and ER24 to examine whether window-specific thresholds materially changed discrimination. Multivariable logistic regression was used to estimate adjusted odds ratios (ORs) for ER12, and multivariable Cox proportional-hazards models were used to estimate adjusted hazard ratios (HRs) for DFS and OS, adjusting for age, sex, TNM stage, tumor size, differentiation, resection margin status, perineural invasion, vascular invasion, CA19-9, chemotherapy, and targeted therapy; the CA125-LMR score was entered as a continuous 0–2 variable, with effect estimates expressed per 1-point increase. Internal validation was performed using bootstrap resampling (1,000 iterations), in which the Youden-derived cutoffs for CA125 and LMR were re-estimated within each resample before constructing the score, and the optimism-corrected AUC was reported. Calibration of the multivariable model was assessed by the calibration intercept, the calibration slope, and the Brier score, with optimism correction by bootstrap validation (1,000 resamples). Clinical utility was evaluated by decision-curve analysis, and the incremental value of the CA125-LMR score over LMR alone was quantified by the continuous net reclassification improvement (NRI) and the integrated discrimination improvement (IDI). A Fine–Gray competing-risk model, with recurrence as the event of interest and non-recurrent death as the competing event, was fitted over the full follow-up. Additional sensitivity analyses were performed by stratifying according to chemotherapy status, by excluding patients with stage IV disease, and by restricting the analysis to patients with CA19-9 values within the normal range (≤37 U/mL). An exploratory phenotype analysis cross-classified patients by the fixed CA125 and LMR cutoffs into four groups and compared observed recurrence proportions at each window.

Pairwise DeLong comparisons were prespecified for the CA125-LMR score vs. the CA19-9-LMR score, CA125 alone, and LMR alone because these directly addressed the study hypothesis. Complete pairwise comparisons are reported in Supplementary Table S2. For the nine hypothesis-focused comparisons (three comparators at three recurrence windows), Holm–Bonferroni correction was applied to control the family-wise error rate, and false-discovery-rate (Benjamini–Hochberg) adjusted *P* values are additionally reported in Supplementary Table S7. Exact *p* values and effect sizes are emphasized.

## Results

### Patient characteristics according to 12-month recurrence

A total of 1,618 patients were included; 779 (48.1%) developed recurrence within 12 months and 839 (51.9%) did not. Baseline characteristics are shown in Table 1. Compared with patients without ER12, those with ER12 had higher median CA125, CA19-9, CEA, and C-reactive protein values and lower median LMR and PNI values. ER12 was also more frequent in patients with stage III–IV disease (particularly stage IV), larger tumors, and poorly differentiated tumors. Sex distributions were similar between groups.

Median CA125 was 50.680 U/mL in the ER12 group and 20.880 U/mL in the non-ER12 group; the corresponding CA19-9 values were 130.200 and 47.880 U/mL. Median LMR was lower in the ER12 group (3.452 vs. 5.948), whereas C-reactive protein was higher (10.670 vs. 4.740) and albumin was lower (39.600 vs. 40.700).

### Predictive performance for 12-month recurrence

For ER12, the CA125-LMR score had an AUC of 0.742 (95% CI, 0.720–0.764), numerically the highest among the evaluated markers. The AUCs were 0.735 (95% CI, 0.711–0.759) for LMR, 0.698 (95% CI, 0.673–0.724) for CA125, 0.692 (95% CI, 0.668–0.715) for the CA19-9-LMR score, and 0.602 (95% CI, 0.574–0.629) for CA19-9. At the selected threshold, the CA125-LMR score had 91.4% sensitivity and 44.3% specificity (Table 2; Figure 1).

**Table 2:** Predictive performance of biomarkers for 12-month recurrence.

Marker	Cutoff	AUC	95% CI	Sensitivity (%)	Specificity (%)	*P* value
CA19-9	59.385	0.602	0.574–0.629	63.8	53.9	<0.001
CA125	44.760	0.698	0.673–0.724	53.5	77.0	<0.001
CEA	3.725	0.671	0.645–0.697	66.1	60.5	<0.001
LMR	5.764	0.735	0.711–0.759	86.0	52.0	<0.001
PNI	47.625	0.593	0.566–0.621	56.6	58.4	<0.001
NLR	2.468	0.631	0.604–0.658	67.3	53.2	<0.001
PLR	136.864	0.554	0.526–0.582	56.4	53.2	<0.001
CA19-9-LMR score	1.5	0.692	0.668–0.715	56.0	71.9	<0.001
CA125-LMR score	0.5	0.742	0.720–0.764	91.4	44.3	<0.001

AUC, area under the receiver operating characteristic curve; CI, confidence interval. *P* values are from two-sided tests of AUC against the null value of 0.5.

**Figure 1:**
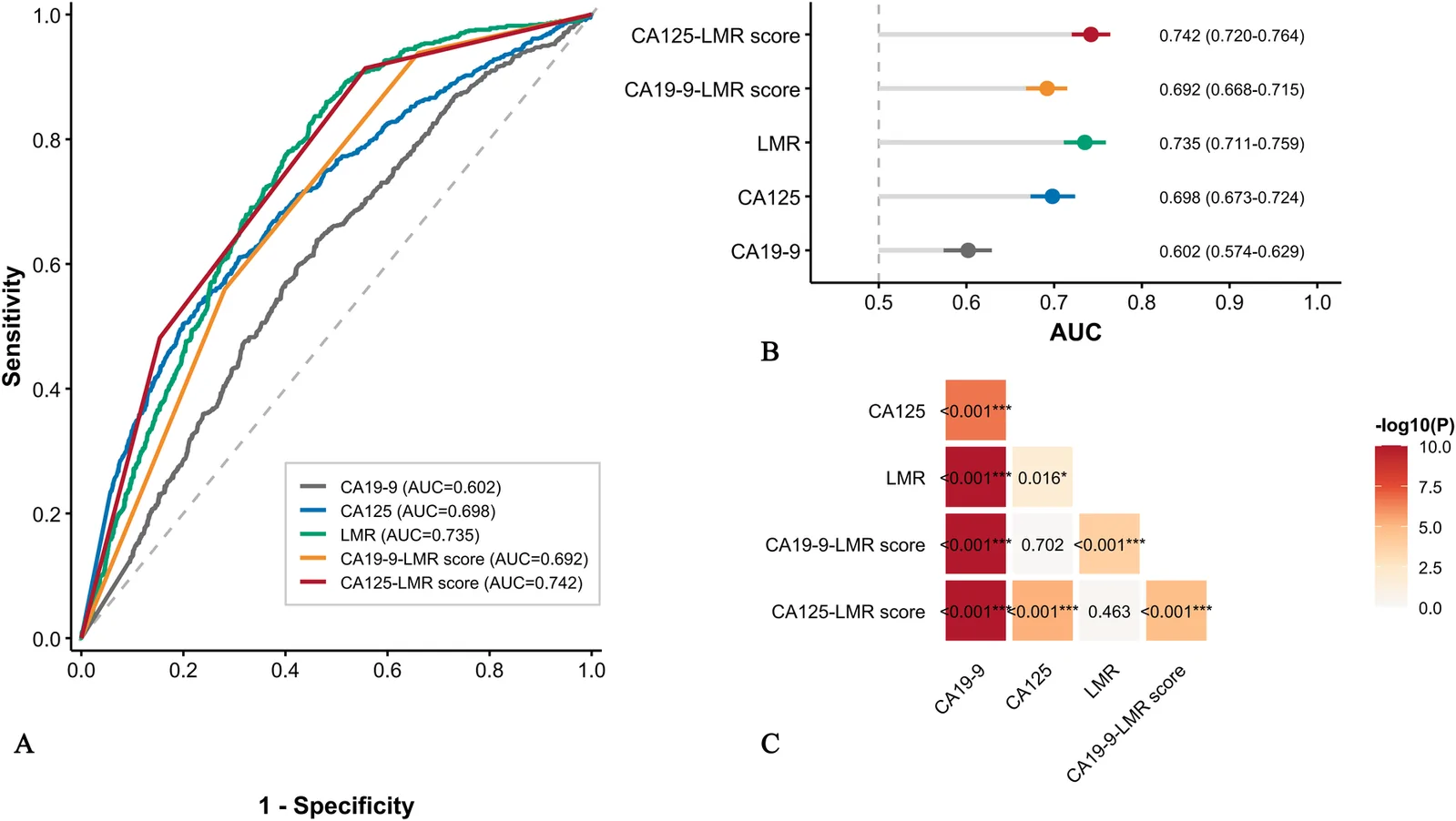
Predictive performance for 12-month recurrence in 1,618 patients (779 ER12 events). **(A)** Receiver operating characteristic curves for CA19-9, CA125, LMR, the CA19-9-LMR score, and the CA125-LMR score. **(B)** Areas under the curve (AUCs) with 95% confidence intervals. **(C)** Pairwise DeLong comparison heatmap; displayed values are two-sided *p* values. ER12, recurrence within 12 months; CA19-9, carbohydrate antigen 19-9; CA125, carbohydrate antigen 125; LMR, lymphocyte-to-monocyte ratio.

The CA125-LMR score had a larger AUC than CA125 alone (*p* < 0.001) and the CA19-9-LMR score (*p* < 0.001), whereas its AUC did not differ from that of LMR alone (*p* = 0.463). Thus, the combined score improved discrimination relative to CA125 and the CA19-9-LMR comparator, but the data do not establish superiority over LMR.

The ROC threshold for the 0–2 score was 0.5, classifying any patient with at least one abnormal component as marker-positive. This threshold accounts for the high sensitivity and modest specificity and indicates that the score is better interpreted as a sensitivity-oriented risk-stratification tool than as a diagnostic test for recurrence.

### Time-window-specific changes in predictive performance

ER6, ER12, and ER24 occurred in 401 (24.8%), 779 (48.1%), and 1,077 (66.6%) patients, respectively. For ER6, the CA125-LMR score had an AUC of 0.694 (95% CI, 0.668–0.719); for ER12, its AUC was 0.742 (95% CI, 0.720–0.764). At 24 months, LMR alone had the highest AUC (0.835; 95% CI, 0.812–0.857), followed by the CA125-LMR score (0.809; 95% CI, 0.787–0.830) (Table 3; Figure 2).

**Table 3:** Predictive performance across 6-, 12-, and 24-month recurrence windows.

Window	Marker	AUC	95% CI	Sensitivity (%)	Specificity (%)	Rank
6-month recurrence	CA125-LMR score	0.694	0.668–0.719	51.9	75.7	1
6-month recurrence	LMR	0.686	0.658–0.713	81.5	48.0	2
6-month recurrence	CA125	0.666	0.635–0.697	58.9	68.0	3
6-month recurrence	CA19-9-LMR score	0.644	0.618–0.671	58.1	63.9	4
6-month recurrence	CA19-9	0.588	0.556–0.620	63.1	51.4	5
12-month recurrence	CA125-LMR score	0.742	0.720–0.764	91.4	44.3	1
12-month recurrence	LMR	0.735	0.711–0.759	86.0	52.0	2
12-month recurrence	CA125	0.698	0.673–0.724	53.5	77.0	3
12-month recurrence	CA19-9-LMR score	0.692	0.668–0.715	56.0	71.9	4
12-month recurrence	CA19-9	0.602	0.574–0.629	63.8	53.9	5
24-month recurrence	LMR	0.835	0.812–0.857	85.7	71.9	1
24-month recurrence	CA125-LMR score	0.809	0.787–0.830	90.8	62.8	2
24-month recurrence	CA19-9-LMR score	0.786	0.764–0.809	94.1	49.7	3
24-month recurrence	CA125	0.711	0.685–0.737	62.2	71.7	4
24-month recurrence	CA19-9	0.649	0.621–0.678	66.4	60.6	5

The fixed ER12-derived cutoffs were applied at all three recurrence windows. Rank was determined by descending AUC within each window.

**Figure 2:**
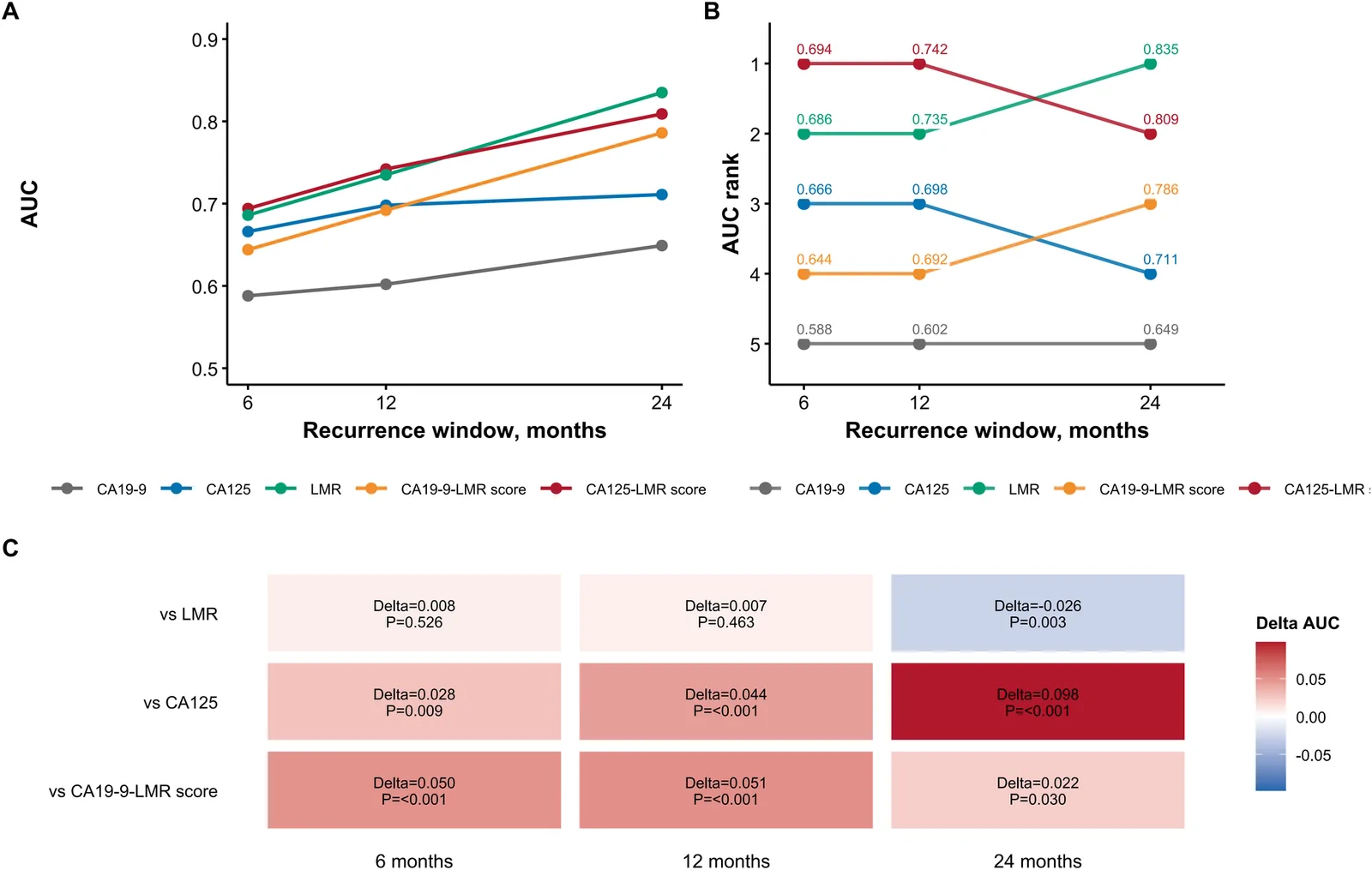
Time-window-specific predictive performance in 1,618 patients. ER6, ER12, and ER24 comprised 401, 779, and 1,077 recurrence events, respectively. **(A)** AUC trajectories for the five core markers across recurrence within 6, 12, and 24 months. **(B)** AUC-rank trajectories, where rank 1 indicates the highest AUC within a recurrence window. **(C)** Focused pairwise DeLong comparisons of the CA125-LMR score with LMR, CA125, and the CA19-9-LMR score. Delta AUC was calculated as the AUC of the CA125-LMR score minus that of the comparator; displayed *p* values are two-sided and unadjusted; Holm–Bonferroni-adjusted *p* values for the nine hypothesis-focused comparisons are reported in Supplementary Table S7. AUC, area under the receiver operating characteristic curve; ER6/ER12/ER24, recurrence within 6/12/24 months.

Compared with the CA19-9-LMR score, the CA125-LMR score had larger AUCs at 6 months (*Δ*AUC, 0.050; *p* < 0.001), 12 months (*Δ*AUC, 0.051; *p* < 0.001), and 24 months (*Δ*AUC, 0.022; *p* = 0.030); after Holm–Bonferroni correction for the nine hypothesis-focused comparisons, the 24-month difference vs. the CA19-9-LMR score was no longer statistically significant (adjusted *P* = 0.089). It also showed higher AUCs than CA125 alone at all three windows. By contrast, it did not differ from LMR at 6 months (*p* = 0.526) or 12 months (*p* = 0.463) and performed less well than LMR at 24 months (*Δ*AUC, −0.026; *p* = 0.003).

The relative ranking changed with the recurrence horizon. The CA125-LMR score consistently showed higher AUCs than CA19-9 and the CA19-9-LMR score, whereas LMR became the strongest discriminator at 24 months. This pattern indicates that the added discrimination from CA125 was concentrated in the earlier windows.

### CA125-LMR score stratifies disease-free survival and observed recurrence risk

The CA125-LMR score separated patients into three groups: score 0 (*n* = 439), score 1 (*n* = 675), and score 2 (*n* = 504). Kaplan–Meier analysis showed progressively poorer DFS across increasing score groups (log-rank *p* < 0.001). This time-to-event pattern was mirrored by observed recurrence rates. At 6 months, recurrence rates were 6.2%, 24.6%, and 41.3% for score 0, 1, and 2, respectively. At 12 months, the corresponding rates were 15.3%, 49.9%, and 74.4%. At 24 months, they were 22.6%, 77.0%, and 90.9%. These gradients support the score as a simple clinical stratification tool rather than only a binary diagnostic cutoff (Figure 3; Supplementary Table S3).

**Figure 3:**
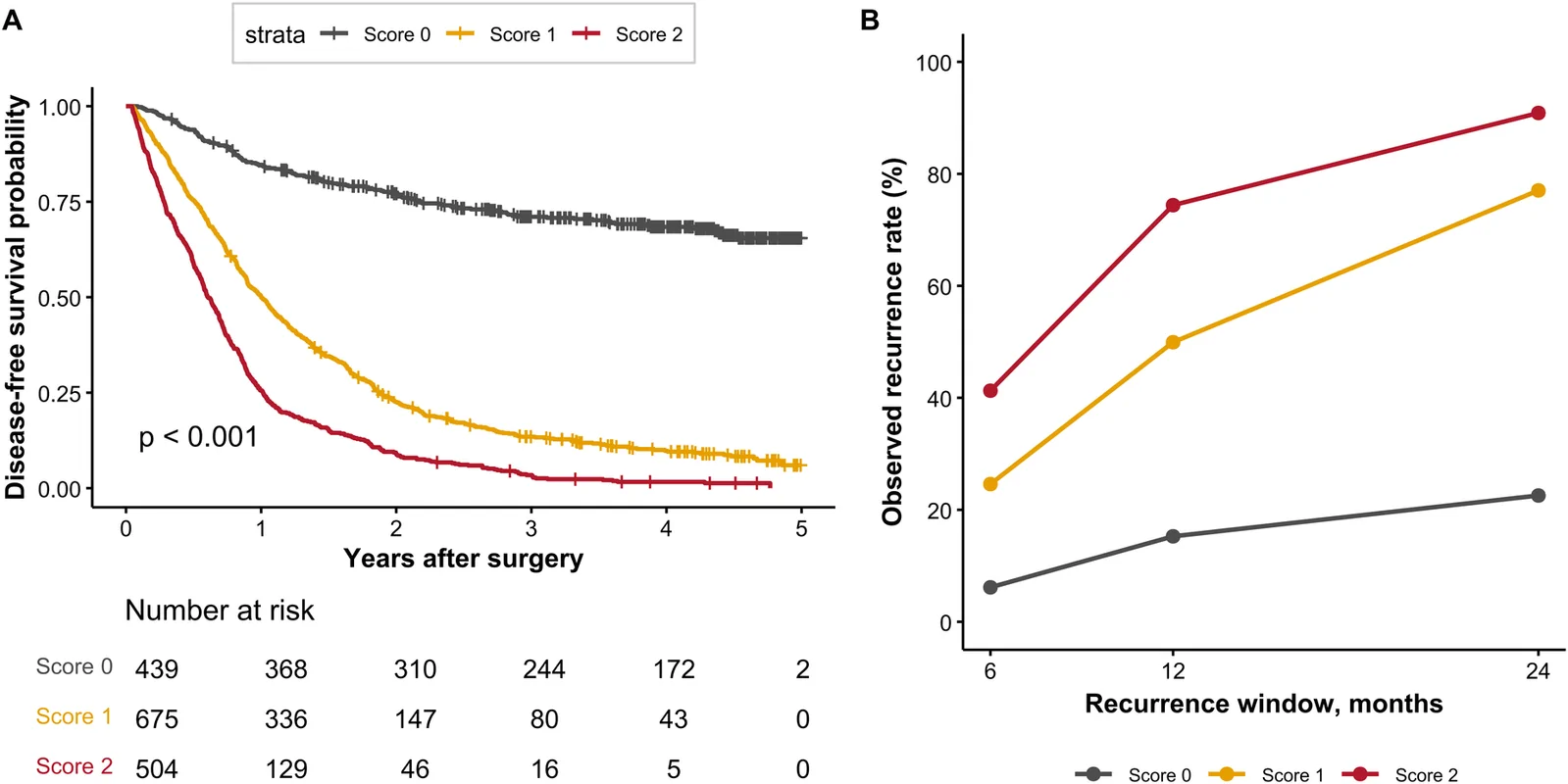
CA125-LMR score stratifies disease-free survival and observed recurrence risk. **(A)** Kaplan–Meier curves for disease-free survival in score 0 (*n* = 439), score 1 (*n* = 675), and score 2 (*n* = 504). Tick marks indicate censored observations, and the risk table reports the number at risk. Groups were compared using a two-sided log-rank test. **(B)** Observed recurrence rates at 6, 12, and 24 months according to score group. CA125, carbohydrate antigen 125; DFS, disease-free survival; LMR, lymphocyte-to-monocyte ratio.

The absolute risk gradients were substantial: by 12 months, recurrence was observed in 15.3%, 49.9%, and 74.4% of score 0, 1, and 2 groups, respectively. These descriptive differences support further evaluation of the score for risk communication and study enrichment; they do not by themselves justify changes in surveillance or treatment.

### Supplementary analyses

Sensitivity analyses showed that recalculating CA125 and LMR cutoffs separately for ER6, ER12, and ER24 did not meaningfully improve the AUC compared with the fixed ER12-derived cutoff approach. For ER6, the window-specific score had an AUC of 0.696 compared with 0.694 for the fixed score (*p* = 0.675). For ER12, the AUCs were identical (0.742), and for ER24, the AUC difference was negligible (0.809 for both approaches, *p* = 0.949). These findings support the stability and interpretability of the fixed ER12-derived CA125-LMR score (Supplementary Table S1). In multivariable models adjusting for age, sex, TNM stage, tumor size, differentiation, resection margin status, perineural invasion, vascular invasion, CA19-9, chemotherapy, and targeted therapy, the associations of the CA125-LMR score with ER12 and DFS remained consistent with the unadjusted findings (Supplementary Table S5). When stratified by chemotherapy status, the score retained its discriminative ability in both subgroups [ER12 AUC, 0.789 without chemotherapy [*n* = 202] and 0.739 with chemotherapy [*n* = 1,416]; Supplementary Table S6]. Bootstrap internal validation (1,000 resamples, with the Youden-derived cutoffs re-estimated within each resample) yielded an optimism-corrected AUC of 0.734 (apparent, 0.742; optimism, 0.009) and corrected calibration estimates (calibration intercept, 0.001; calibration slope, 0.959; Brier score, 0.184) (Supplementary Figure S3; Supplementary Table S8). Decision-curve analysis showed positive net benefit over the treat-none strategy across all thresholds and over the treat-all strategy at thresholds of 20% or higher (Supplementary Figures S3, S4). In a Fine–Gray competing-risk model, each 1-point increase in the score was associated with a subdistribution hazard ratio of 2.25 (95% CI, 2.06–2.45; *p* < 0.001) for recurrence. After excluding the 126 patients with stage IV disease, the AUC was 0.743 and the graded ER12 rates were unchanged (14.8%, 50.4%, 73.7%; Supplementary Table S8). In patients with CA19-9 within the normal range (≤37 U/mL, *n* = 650), the score retained an AUC of 0.791 (95% CI, 0.758–0.824), with graded ER12 rates of 9.9%, 44.8%, and 72.7%.

In the four-phenotype analysis, patients with low CA125 and high LMR had the lowest recurrence rates at each window. At 24 months, recurrence was more frequent in the LMR-low-only phenotype than in the CA125-high-only phenotype (81.4% vs. 53.8%) (Supplementary Figure S2; Supplementary Table S4). This association is consistent with greater late-window discrimination by LMR, although mechanistic explanations cannot be inferred from the observational data. Higher CA125-LMR scores were also associated with poorer overall survival (Supplementary Figure S1).

Together, the sensitivity and phenotype analyses showed that the principal findings were not materially altered by recalculating cutoffs and that CA125 and LMR contributed differently across recurrence windows. These analyses are exploratory and require validation in independent cohorts.

## Discussion

In this cohort of 1,618 patients undergoing pancreatic cancer surgery, an ER12-derived CA125-LMR score stratified recurrence risk across 6-, 12-, and 24-month windows. For the primary ER12 endpoint, the score showed higher discrimination than CA125 alone and the CA19-9-LMR score but was comparable to LMR alone. At 24 months, LMR performed better than the combined score. These findings indicate that biomarker discrimination depends on the recurrence horizon and that the principal value of adding CA125 was observed in the earlier windows.

Postoperative recurrence is clinically heterogeneous. Very early recurrence may be associated with occult metastatic disease and aggressive tumor features, whereas later recurrence reflects additional influences from adjuvant treatment exposure and host condition (41). The observed changes in AUC therefore support reporting a clearly defined prediction horizon rather than treating recurrence as a time-invariant endpoint. The present observational analysis, however, cannot determine the biological mechanisms underlying these temporal differences.

Our study extends the tumor-marker–inflammation framework reported by Yugawa et al. (23) by evaluating CA125 and by examining three recurrence windows. The CA125-LMR score had greater discrimination than the CA19-9-LMR score at each window. This finding supports CA125 as a complementary candidate for recurrence-risk stratification, particularly when CA19-9 may be affected by biliary obstruction or Lewis antigen status (24).

CA19-9 remains the established serum biomarker in pancreatic cancer and should not be displaced on the basis of this single retrospective cohort. The CA125-LMR score should instead be viewed as an investigational complementary approach. Its advantage is practical interpretability; its limitations include cohort-derived cutoffs, modest specificity, and lack of external validation.

Prior studies have associated CA125 with metastatic burden and prognosis after pancreatic cancer resection (25, 26), and MUC16-related biology provides a plausible basis for these associations (27, 28). CA125 has also appeared in recent prognostic and early-recurrence models (9, 21, 29). In our data, CA125 alone showed moderate discrimination, while its combination with LMR produced clear risk gradients. These findings are associative and do not establish a causal role for CA125.

LMR remained a strong standalone marker, particularly at 24 months, consistent with reports linking inflammatory and nutritional indices to pancreatic cancer outcomes (30–38). Low LMR may reflect lymphopenia, monocytosis, or systemic inflammatory status, but this study did not directly measure immune function. The higher 24-month recurrence rate in the LMR-low-only group than in the CA125-high-only group provides descriptive support for the time-window pattern.

Other inflammation- and nutrition-based measures—including NLR, PLR, C-reactive protein-based indices, PNI, and physiobiological grading systems—have also been evaluated in resectable pancreatic cancer (13, 15, 22, 30, 36–38). Our findings do not demonstrate mechanistic or prognostic superiority of LMR over all alternatives; they identify LMR as the strongest discriminator among the evaluated markers at the 24-month horizon.

The score uses routine preoperative tests and yields an interpretable 0–2 classification. Its high sensitivity but modest specificity at the ER12 threshold suggests potential value for identifying patients unlikely to belong to a low-risk group. Before clinical use, however, the score should be externally validated, calibrated, and assessed for incremental value beyond stage, pathology, treatment, and standard biomarkers.

The findings may inform the design of future validation studies. Such studies should prespecify the recurrence horizon, use contemporary treatment cohorts, report calibration and clinical utility, and compare the score with clinical models rather than biomarkers alone. Decision-curve analysis and evaluation in CA19-9 non-producers may further clarify whether the score adds actionable information.

Strengths include the large cohort, direct comparison with the published CA19-9-LMR framework, formal pairwise AUC testing, use of fixed cutoffs across time windows, and phenotype analyses that improve interpretability. The results are consistent across the main and cutoff-sensitivity analyses.

Several limitations merit emphasis. First, the retrospective design permits selection bias and residual confounding. Second, the cutoffs and performance estimates were derived and internally validated in the same cohort; although bootstrap-based optimism correction indicated minimal optimism (0.009), external validation in independent cohorts is still required before clinical use. Third, although multivariable adjustment, calibration, and decision-curve analyses were additionally performed, the score was evaluated primarily as a discrimination and risk-stratification tool rather than against a comprehensive multivariable clinical prediction model. Fourth, systemic treatment was recorded only as binary indicators of any chemotherapy or targeted therapy, without information on neoadjuvant vs. adjuvant intent, specific regimens, treatment completion, or the timing of treatment relative to biomarker assessment, which may introduce residual confounding. Fifth, prespecified pre-analytical rules were applied at data collection (preoperative sample closest to surgery; post-drainage values after biliary drainage; exclusion of samples obtained during acute infection or overt inflammation). Individual-level details—sampling-to-surgery interval, cholestasis status, corticosteroid exposure, and timing of recent systemic therapy—were not retained, so residual pre-analytical variability cannot be excluded; Lewis phenotypes for identifying CA19-9 non-producers were also unavailable. Sixth, the modality used to confirm each recurrence was not recorded per event, so the primary analyses could not be restricted to imaging- or pathology-confirmed recurrences.

In conclusion, the CA125-LMR score provided simple, interpretable recurrence-risk stratification after pancreatic cancer surgery, with its clearest performance in the 6- and 12-month windows. LMR alone showed greater discrimination at 24 months. Independent validation and assessment against comprehensive clinical models are required before the score can inform surveillance or treatment decisions.

## Data Availability

The datasets presented in this article are not readily available because the datasets contain individual-level clinical and follow-up information and are not publicly available due to patient privacy, confidentiality, ethical approval, and institutional data-governance restrictions. Requests for access may be directed to the corresponding author and will be considered in accordance with applicable ethical and institutional requirements. Requests to access the datasets should be directed to Qingfeng Tian, zzutqf@126.com.
