# Supplementary material for: A combined carbohydrate antigen 125 and lymphocyte-to-monocyte ratio score stratifies time-window-specific recurrence after pancreatic cancer surgery
Source: Front Med (Lausanne). 2026 Oct 8;13:1971334. doi: 10.3389/fmed.2026.1971334 (PMC13689726; doi:10.3389/fmed.2026.1971334)
Supplement: Supplementary file 1 [file Supplementaryfile1.pdf]

Supplementary Table S1. Sensitivity analysis using time-window-specific CA125 and LMR cutoffs.

Recurrence window	CA125 cutoff, U/mL	LMR cutoff	Fixed-cutoff AUC	95% CI	Window-specific AUC	95% CI	ΔAUC	DeLong p value	Window-specific score distribution
6-month recurrence	43.79	5.053	0.694	0.668-0.719	0.696	0.67-0.722	0.002	0.675	Score 0: 511; Score 1: 629; Score 2: 478
12-month recurrence	44.76	5.764	0.742	0.72-0.764	0.742	0.72-0.764	0	1.000	Score 0: 439; Score 1: 675; Score 2: 504
24-month recurrence	28.045	5.777	0.809	0.787-0.83	0.809	0.787-0.831	0	0.949	Score 0: 377; Score 1: 584; Score 2: 657

The primary analysis used the fixed ER12-derived cutoffs of CA125 44.760 U/mL and LMR 5.764. Window-specific cutoffs were selected using the Youden index. ΔAUC was calculated as the window-specific-score AUC minus the fixed-score AUC. AUC, area under the receiver operating characteristic curve; CA125, carbohydrate antigen 125; CI, confidence interval; ER12, recurrence within 12 months; LMR, lymphocyte-to-monocyte ratio.

Supplementary Table S2. All pairwise DeLong comparisons across recurrence windows.

Recurrence window	Marker 1	Marker 2	AUC 1	AUC 2	ΔAUC	Two-sided p value
6-month recurrence	CA19-9	CA125	0.588	0.666	-0.078	<0.001
6-month recurrence	CA19-9	LMR	0.588	0.686	-0.098	<0.001
6-month recurrence	CA19-9	CA19-9-LMR score	0.588	0.644	-0.056	<0.001
6-month recurrence	CA19-9	CA125-LMR score	0.588	0.694	-0.106	<0.001
6-month recurrence	CA125	LMR	0.666	0.686	-0.020	0.266
6-month recurrence	CA125	CA19-9-LMR score	0.666	0.644	0.022	0.273
6-month recurrence	CA125	CA125-LMR score	0.666	0.694	-0.028	0.009

6-month recurrence	LMR	CA19-9-LMR score	0.686	0.644	0.042	0.003
6-month recurrence	LMR	CA125-LMR score	0.686	0.694	-0.008	0.526
6-month recurrence	CA19-9-LMR score	CA125-LMR score	0.644	0.694	-0.050	<0.001
12-month recurrence	CA19-9	CA125	0.602	0.698	-0.097	<0.001
12-month recurrence	CA19-9	LMR	0.602	0.735	-0.133	<0.001
12-month recurrence	CA19-9	CA19-9-LMR score	0.602	0.692	-0.090	<0.001
12-month recurrence	CA19-9	CA125-LMR score	0.602	0.742	-0.141	<0.001
12-month recurrence	CA125	LMR	0.698	0.735	-0.037	0.016
12-month recurrence	CA125	CA19-9-LMR score	0.698	0.692	0.006	0.702
12-month recurrence	CA125	CA125-LMR score	0.698	0.742	-0.044	<0.001
12-month recurrence	LMR	CA19-9-LMR score	0.735	0.692	0.043	<0.001
12-month recurrence	LMR	CA125-LMR score	0.735	0.742	-0.007	0.463
12-month recurrence	CA19-9-LMR score	CA125-LMR score	0.692	0.742	-0.051	<0.001
24-month recurrence	CA19-9	CA125	0.649	0.711	-0.062	0.001
24-month recurrence	CA19-9	LMR	0.649	0.835	-0.186	<0.001
24-month recurrence	CA19-9	CA19-9-LMR score	0.649	0.786	-0.137	<0.001
24-month recurrence	CA19-9	CA125-LMR score	0.649	0.809	-0.159	<0.001
24-month recurrence	CA125	LMR	0.711	0.835	-0.124	<0.001
24-month recurrence	CA125	CA19-9-LMR score	0.711	0.786	-0.075	<0.001
24-month recurrence	CA125	CA125-LMR score	0.711	0.809	-0.098	<0.001

24-month recurrence	LMR	CA19-9-LMR score	0.835	0.786	0.048	<0.001
24-month recurrence	LMR	CA125-LMR score	0.835	0.809	0.026	0.003
24-month recurrence	CA19-9-LMR score	CA125-LMR score	0.786	0.809	-0.022	0.030

ΔAUC was calculated as AUC 1 minus AUC 2. P values are from paired, two-sided DeLong tests; no adjustment for multiple comparisons was applied. Full-precision numeric p values are retained in the accompanying Supplementary Data workbook. AUC, area under the receiver operating characteristic curve; CA19-9, carbohydrate antigen 19-9; CA125, carbohydrate antigen 125; LMR, lymphocyte-to-monocyte ratio.

Supplementary Table S3. Observed recurrence rates according to CA125-LMR score.

Recurrence window	Score 0, events/n (%)	Score 1, events/n (%)	Score 2, events/n (%)
6-month recurrence	27/439 (6.2%)	166/675 (24.6%)	208/504 (41.3%)
12-month recurrence	67/439 (15.3%)	337/675 (49.9%)	375/504 (74.4%)
24-month recurrence	99/439 (22.6%)	520/675 (77%)	458/504 (90.9%)

Values are events/n (%). Score 0: CA125 ≤44.760 U/mL and LMR ≥5.764; score 1: one abnormal component; score 2: CA125 >44.760 U/mL and LMR <5.764. Rates are descriptive observed proportions at each recurrence window. CA125, carbohydrate antigen 125; LMR, lymphocyte-to-monocyte ratio.

Supplementary Table S4. Observed recurrence rates according to CA125-LMR phenotype.

Recurrence window	CA125 low + LMR high	CA125 high only	LMR low only	CA125 high + LMR low
6-month recurrence	27/439 (6.2%)	23/106 (21.7%)	143/569 (25.1%)	208/504 (41.3%)
12-month recurrence	67/439 (15.3%)	42/106 (39.6%)	295/569 (51.8%)	375/504 (74.4%)
24-month recurrence	99/439 (22.6%)	57/106 (53.8%)	463/569 (81.4%)	458/504 (90.9%)

Values are events/n (%). “CA125 high” denotes CA125 >44.760 U/mL, and “LMR low” denotes LMR <5.764. Rates are descriptive observed proportions at each recurrence window. CA125, carbohydrate antigen 125; LMR, lymphocyte-to-monocyte ratio.

Supplementary Table S5. Association of the CA125-LMR score with 12-month recurrence (logistic regression) and with disease-free and overall survival (Cox regression).

Model	ER12 (logistic regression)		DFS (Cox regression)		OS (Cox regression)	
	OR (95% CI)	Pvalue	HR (95% CI)	P value	HR (95% CI)	P value
Model1	3.878(3.317-4.555)	<0.001	2.492(2.304-2.697)	<0.001	2.673(2.467-2.897)	<0.001
Model2	3.611(3.026-4.331)	<0.001	2.319(2.128-2.527)	<0.001	2.433(2.230-2.655)	<0.001

Model1:CA125_LMR_score;

Model2:CA125_LMR_score,Age,Sex,TNM stage,Tumor size,Differentiation,Resection margin status，Perineural invasion，Vascular invasion，CA19-9，Chemotherapy，Targeted therapy

Supplementary Table S6. Discriminative performance of the CA125-LMR score for 12-month recurrence (ER12) according to chemotherapy status.

Chemotherapy status	N	ER12, n (%)	AUC
Without Chemotherapy	202	115 (56.9%)	0.789
With Chemotherapy	1416	664 (46.9%)	0.739

ER12, recurrence within 12 months after surgery; AUC, area under the receiver operating characteristic curve of the CA125-LMR score for ER12.

Supplementary Table S7. Pairwise DeLong comparisons of the CA125-LMR score with LMR, CA125, and the CA19-9-LMR score for recurrence within 6, 12, and 24 months

Recurrence window	Comparison	Raw P value	Holm-adjusted P value	FDR-adjusted P value
6-month recurrence	vs LMR	0.526	0.925	0.526
6-month recurrence	vs CA125	0.009	0.037	0.014
6-month recurrence	vs CA19-9-LMR	<0.001	0.004	0.001
12-month recurrence	vs LMR	0.463	0.925	0.520
12-month recurrence	vs CA125	<0.001	<0.001	<0.001
12-month recurrence	vs CA19-9-LMR	<0.001	<0.001	<0.001
24-month recurrence	vs LMR	0.003	0.014	0.005
24-month recurrence	vs CA125	<0.001	<0.001	<0.001
24-month recurrence	vs CA19-9-LMR	0.030	0.089	0.038

All comparisons are of the CA125-LMR score versus the indicated comparator using DeLong tests (nine hypothesis-focused comparisons). Holm, Holm–Bonferroni multiplicity correction; FDR, false discovery rate correction (Benjamini–Hochberg).

Supplementary Table S8. Internal validation and sensitivity analyses of the CA125-LMR score and the multivariable model for 12-month recurrence.

Metric	Apparent value	Bootstrap-corrected value
**Discrimination (ER12)**		
AUC	0.742	0.734
Optimism	—	0.009
**Calibration (multivariable model)**		
Calibration intercept	0	0.001
Calibration slope	1	0.959
Brier score	0.179	0.184
**Sensitivity analysis (excluding stage IV disease)**		
AUC (n = 1,492)	—	0.743 (95% CI, 0.718–0.768)
ER12 rate, score 0 / 1 / 2	—	14.8% / 50.4% / 73.7%

Bootstrap validation with 1,000 resamples; the Youden-derived cutoffs for CA125 and LMR were re-estimated within each resample. ER12, recurrence within 12 months; AUC, area under the receiver operating characteristic curve; CI, confidence interval. The calibration model used the linear predictor of the multivariable model as its only covariate.

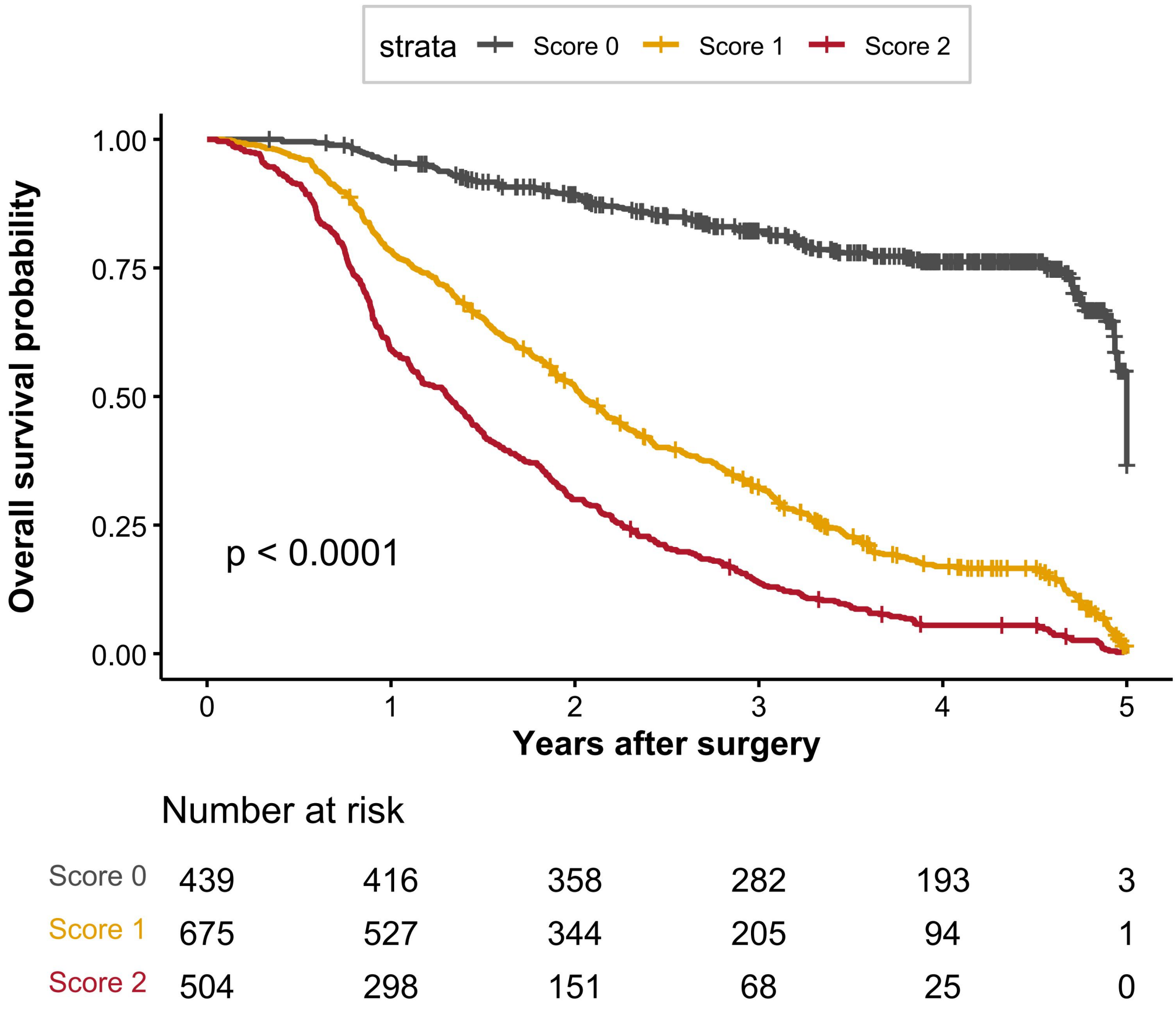

Supplementary Figure S1. Overall survival according to the CA125-LMR score. Kaplan – Meier curves show overall survival for score 0 (n = 439), score 1 (n = 675), and score 2 (n = 504). Tick marks indicate censored observations, and the risk table shows the number at risk. Groups were compared using a two-sided log-rank test ($p < 0.0001$). OS, overall survival; CA125, carbohydrate antigen 125; LMR, lymphocyte-to-monocyte ratio.

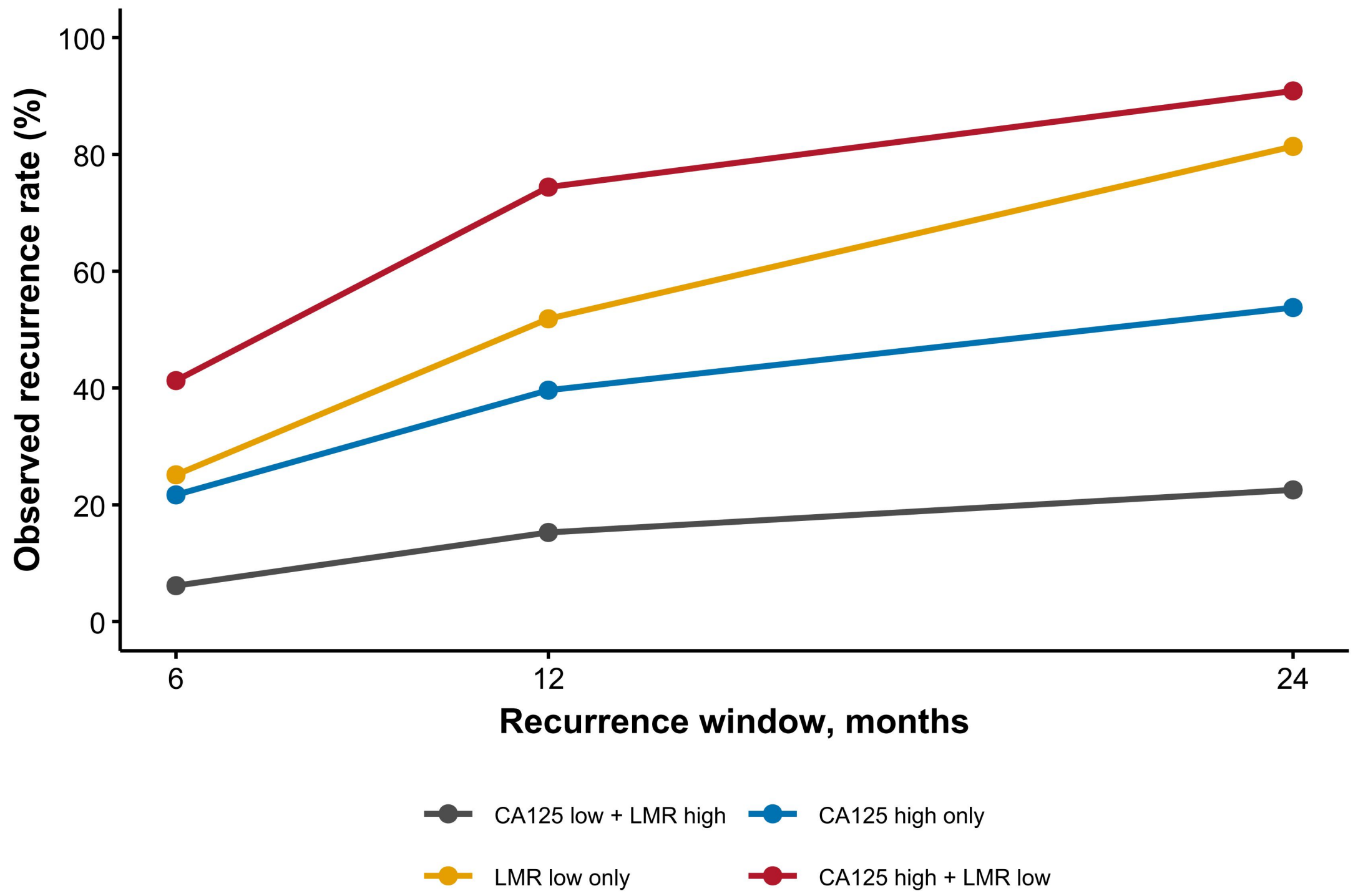

Supplementary Figure S2. Observed recurrence rates according to CA125-LMR phenotype. Rates at 6, 12, and 24 months are shown for CA125 low/LMR high (n = 439), CA125 high only (n = 106), LMR low only (n = 569), and CA125 high/LMR low (n = 504). Phenotypes were defined using the ER12-derived cutoffs of CA125 44.760 U/mL and LMR 5.764. CA125, carbohydrate antigen 125; ER12, recurrence within 12 months; LMR, lymphocyte-to-monocyte ratio.

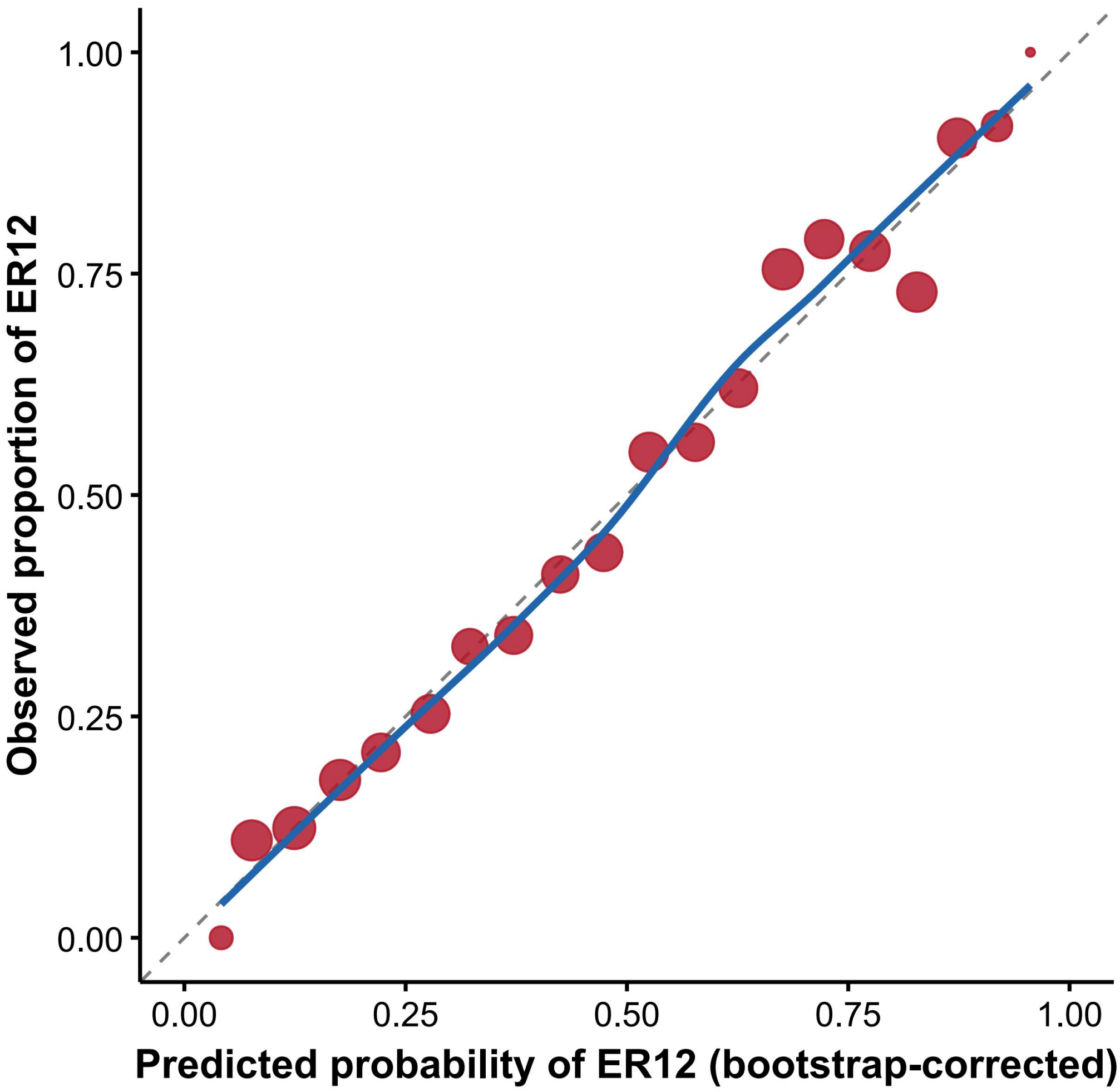

Supplementary Figure S3. Bootstrap-corrected calibration curve for 12-month recurrence. The solid line represents the bias-corrected calibration curve (1,000 bootstrap resamples), and the dashed line indicates ideal calibration. The bootstrap-corrected calibration intercept was 0.001, the calibration slope was 0.959, and the Brier score was 0.184. ER12, recurrence within 12 months.

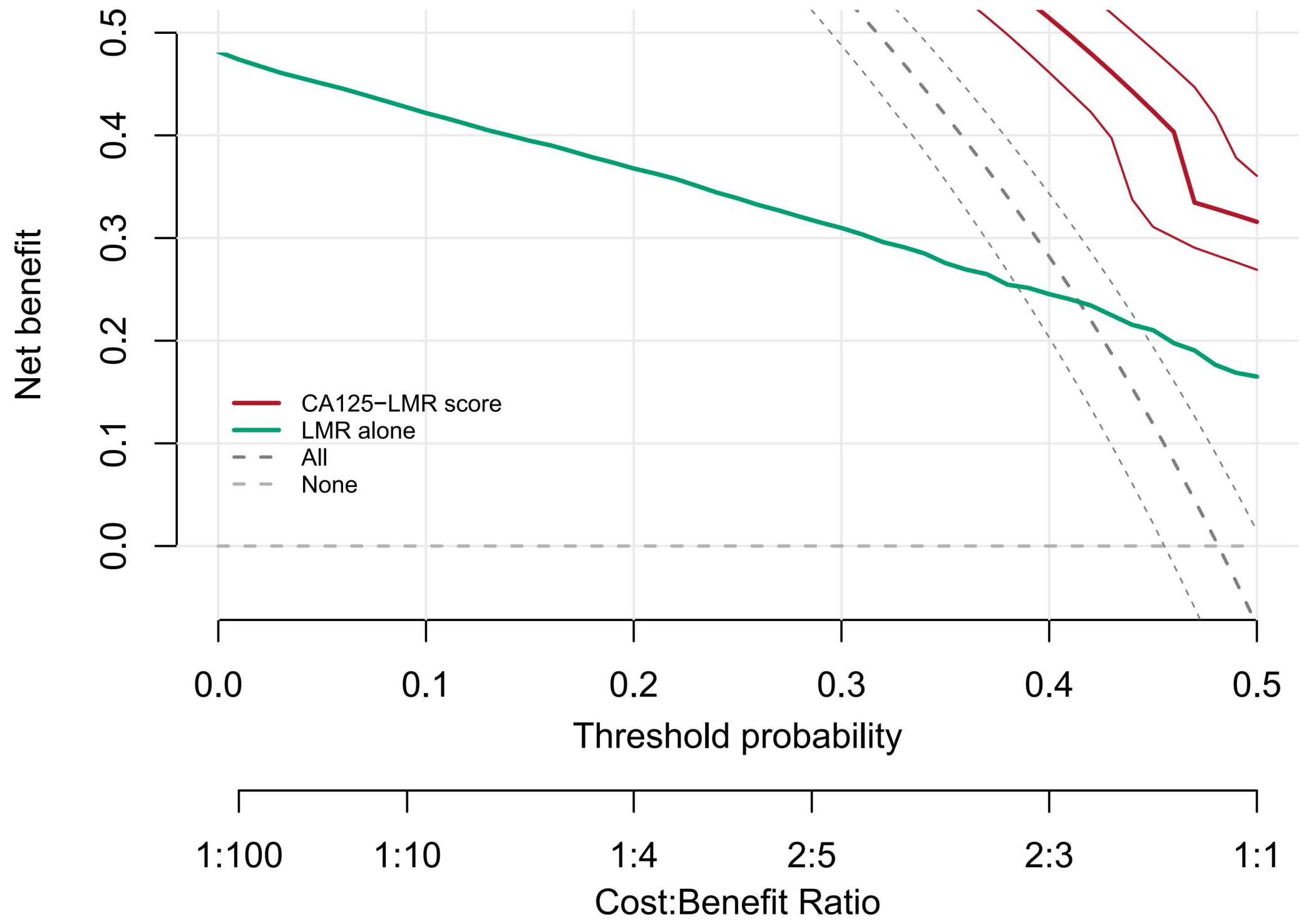

Supplementary Figure S4. Decision-curve analysis for 12-month recurrence. Net benefit is plotted against threshold probability for the CA125-LMR score and LMR alone, together with the treat-all and treat-none reference strategies. The CA125-LMR score showed positive net benefit over the treat-none strategy across all thresholds and over the treat-all strategy at threshold probabilities of 20% or higher. CA125, carbohydrate antigen 125; ER12, recurrence within 12 months; LMR, lymphocyte-to-monocyte ratio.
